# 

**Published:** 1881-02

**Authors:** 


### Table of Contents.

### ORIGINAL COMMUNICATIONS.

A Case of Chloroform Poisoning Treated by Hypodermic Injections of Whisky and Strychine—Recovery. By Robert W. Westmoreland, M.D , Atlanta, Ga........ 641

### REPORTS OF SOCIETIES.

Pathological Society of Philadelphia........ 643
Academy of Medicine and Surgery cf Kentucky........ 668
St. Louis Obstetrical and Gynecological Society........ 675

### SELECTIONS.

" Anæsthesia in Labor."........ 680
Amblyopia from the Abuse of Tobacco and Alcohol........ 682
What Determines the Sex of Children........ 686
Malaria in New England........ 688
On the Treatment of Pneumonia........ 698

### EDITORIAL.

Medical Legislature and Ethical Changes........ 703
Infection........ 704
Meteorological Report for February........ 707

### BIBLIOGRAPHICAL.

The Bacteria........ 705
How a Person Threatened or Afflicted with Bright's Disease Ought to Live........ 706
Hand Book of Systematic Urinary Analysis, Chemical and Microscopical........ 706
Valuable Medical Prescriptions........ 706
Pamphlets Received........ 706

### EXCERPTA.

The Rubber Syringe as a Substitute for the Stomach Pump........ 708
The Etiology of Leprosy........ 709
Medical Experts........ 710
Contraction of the Uterine Orifices in Relation to Dysmenorrhea and Sterility........ 711
As a Commentary on a Certain Class of "Regular" Advertising........ 711
A Peculiar Device........ 711
Symmetrical Neuralgia in Diabetes........ 712
A New Method of Preserving Raw Meat........ 712
Mr. Lister........ 712

